# World Malaria Day — April 25, 2013

**Published:** 2013-04-19

World Malaria Day is commemorated on April 25, the date in 2000 when 44 African leaders met in Abuja, Nigeria, and committed their countries to cutting malaria-related deaths. In the last decade, increased funding and political commitment have led to a scale-up of effective malaria prevention and control interventions, saving approximately 1.1 million lives globally and decreasing malaria mortality by nearly 25% globally and 33% in sub-Saharan Africa (1). Despite these successes, an estimated 660,000 malaria-related deaths occurred worldwide in 2010 (1). For 2013, the theme of World Malaria Day is “Invest in the Future: Defeat Malaria,” which serves as a reminder of the ultimate goal.

CDC supports global malaria efforts through the President’s Malaria Initiative, a U.S. government interagency initiative to reduce malaria incidence and mortality in 19 countries in sub-Saharan Africa and in the Greater Mekong Subregion in Asia. This effort has helped deliver millions of insecticide-treated mosquito nets, antimalarial drugs, and rapid diagnostic test kits to ensure that everyone at risk for malaria has access to life-saving prevention and treatment. In addition, CDC conducts multidisciplinary strategic and applied research globally to increase knowledge about malaria and develop safe, effective interventions that can lead to the elimination and eventual eradication of malaria.

Additional information about CDC’s malaria activities is available at http://www.cdc.gov/malaria.
